# The influence of pre-stenting and drugs on the outcomes of ureteroscopy for kidney and ureteral stone disease: a systematic review and meta-analysis by the EAU Section of Endourology

**DOI:** 10.1007/s00345-025-05848-2

**Published:** 2025-08-12

**Authors:** Steffi Kar Kei Yuen, Daniele Castellani, Theodoros Tokas, Giacomo Maria Pirola, Carlo Giulioni, Jia-Lun Kwok, Mohamed Amine Lakmichi, Takafumi Yanagisawa, Amelia Pietropaolo, Thomas Herrmann, Bhaskar Somani, Vineet Gauhar

**Affiliations:** 1 SH Ho Urology Centre, Department of Surgery, The Chinese University of Hong Kong, Hong Kong, China; 2 European Association of Urology Section of Endourology, Arnhem, The Netherlands; 3 Urology Unit, Azienda Ospedaliero-Universitaria delle Marche, Ancona, Italy; 4 Department of Urology, University General Hospital of Heraklion, University of Crete, Medical School, Heraklion, Greece; 5 Training and Research in Urological Surgery and Technology (T.R.U.S.T.)-Group, Hall in Tirol, Austria; 6 IRCCS Multimedica, Ospedale San Giuseppe, Milano, Italy; 7 Department of Urology, Casa di Cura Villa Igea, Ancona, Italy; 8 Department of Urology, Tan Tock Seng Hospital, Singapore, Singapore; 9 Department of Urology, University Hospital Mohammed the VIth of Marrakesh, Marrakesh, Morocco; 10 Department of Urology, The Jikei University School of Medicine, Tokyo, Japan; 11 Department of Urology, University Hospital Southampton, NHS Trust, Southampton, UK; 12 Department of Urology, Kantonspital Frauenfeld, Spital Thurgau AG, Frauenfeld, Switzerland; 13 Department of Urology, Ng Teng Fong Hospital, Singapore, Singapore

**Keywords:** Alpha-blocker, Kidney stone, Silodosin, Tamsulosin, Pre-stenting, Ureteral, Ureteroscopy

## Abstract

**Introduction:**

Ureteroscopy serves as a minimally invasive surgical treatment option for ureteral and kidney stones but is not without technical challenges. Pre-stenting and medical therapies, such as alpha-blockers, may improve outcomes by facilitating ureteral access sheath placements and reducing complications. This systematic review and meta-analysis aimed to evaluate the effects of pre-stenting and preoperative pharmacological agents on ureteroscopy outcomes.

**Methods:**

We conducted a systematic review and meta-analysis following PRISMA guidelines. A comprehensive literature search was performed across multiple databases, identifying randomized controlled trials comparing ureteroscopy outcomes with and without pre-stenting and pre-ureteroscopy medical therapy. Primary outcomes included failure rates in accessing the upper urinary tract, while secondary outcomes encompassed operative times, complications, and stone-free rates.

**Results:**

The analysis included 16 randomized controlled trials, revealing that pre-stenting and medical therapy significantly reduced failure rates (Relative Ratio 0.44, 95% CI 0.33–0.59, p < 0.001) and operative times (Mean Difference −10.81 min, 95% CI −13.45 to −8.18, p < 0.001). Additionally, there was a higher stone-free rates, lower need for postoperative stenting and fewer complications associated with preoperative ureteral dilation interventions.

**Conclusion:**

Preoperative alpha blockers enhance ureteroscopy success and reduces complications. The evidence supports their use before ureteroscopy for renal and ureteral stones, improving patient outcomes and procedural efficiency. Patients pre-stented for any reason demonstrated significantly improved ureteroscopic access and stone-free rates.

**Supplementary Information:**

The online version contains supplementary material available at 10.1007/s00345-025-05848-2.

## Introduction

Semirigid (sURS) and flexible ureteroscopy (fURS) are among the primary treatments for ureteral and kidney stones [1]. Advances in instrumentation have expanded their implementation in complex cases [2]. Even for experienced surgeons, both semirigid and flexible ureteroscopy (sURS and fURS) pose numerous difficulties. These issues arise from the initial insertion of the instrument and persist throughout its progression, including the placement of a ureteral access sheath (UAS). Nevertheless, instrumentation with ureteroscopes or UAS may cause severe ureteral injury and postoperative ureteral strictures due to excessive buckling force during insertion and intraluminal ureteral compression with ischaemia [3, 4]. Furthermore, unsuccessful procedures lead to additional intervention sessions adding to the psychological and cost burdens to patients. Balloon dilation and sequential ureteral dilators have been utilized to promote primary ureteral access, yet their implementation is not devoid of complications [5, 6].

Preoperative medical therapy targets multiple pathways, including the blockade of alpha-1A and alpha-1D adrenoceptors in the ureter, to facilitate ureteral dilation. Alpha-blockers help improve ureteral access, spontaneous fragment clearance and stent related lower urinary tract symptoms [7–9]. Moreover, aminophylline has been used to relieve spasms and increase treatment success [10]. Finally, ureteral stents are often placed before ureteroscopy to facilitate deployment of UAS when facing a challenging case [11].

This study aimed to systematically evaluate the effects of medical drugs and pre-stenting, compared to placebo or no pre-stenting, on the operative outcomes of sURS or fURS for the treatment of renal and ureteral stones

## Material and methods

We conducted a systematic review and meta-analysis, according to the Preferred Reporting Items for Systematic Reviews and Meta-Analyses (PRISMA) 2020 statement [12], to evaluate the efficacy of using drugs or pre-stenting before ureteroscopy for renal or ureteral stones. The primary objective was to determine how the use of preoperative drugs or pre-stenting affects the failure rate of accessing the upper urinary tract. The secondary outcomes were operative times, intraoperative adverse events and postoperative complications, stone-free rates, and the incidence of ancillary procedures.

### Data sources and searches

With no date limit, a literature search was performed on 3rd November 2024, using PubMed, CENTRAL, Scopus, and Google Scholar. The following terms and Boolean operators were used: (ureteroscopy OR URS OR retrograde intrarenal surgery OR RIRS) AND (pre-stenting OR ureteral stent OR stenting OR preoperative stenting) AND (drugs OR medication OR medical therapy) AND (dilatation OR ureteral dilatation OR balloon dilatation OR ureteral access). The review protocol was registered in PROSPERO with the registration number CRD42024616013.

### Selection criteria

The PICOS (Patient, Intervention, Comparison, Outcome, Study type) model was used to frame and answer the clinical question: P: adults or children with renal/ureteral stones undergoing ureteroscopy; I: any preoperative ureteral dilatation or pre-stenting or drugs for ureteral dilatation; C: pre-op placebo or URS without pre-stenting or drugs; O: primary: access to the renal/ureteral collecting system; secondary: surgical and ureteroscopy times, complications, stone-free rates, ancillary procedures; postoperative stent usage S: prospective and randomized studies.

Studies were included based on PICOS eligibility criteria, with only English-language publications accepted. Animal and preclinical studies, reviews, letters to the editor, case reports, and conference abstracts were excluded. Studies lacking data suitable for meta-analysis were also excluded. Eligible study designs included only prospective randomized trials.

Two independent authors screened all retrieved studies using Covidence systematic review software (Veritas Health Innovation, Melbourne, Australia). A third author resolved any discrepancies. Full texts of the screened articles were selected if deemed relevant to the scope of this review.

### Statistical analysis

Categorical variables were assessed using the Cochran-Mantel-Haenszel Method with the random effect model and reported as Risk Ratio (RR), 95% confidence interval (CI), and p-value. Continuous variables were pooled using the inverse variance of the mean difference (MD) with a random effect, 95% CI, and p-value. Analyses were two-tailed, and the significance was set at p < 0.05 and a 95% CI. RR less than one indicates a lower risk in the experimental group (i.e., pre-ureteroscopy dilation group). A subgroup analysis was performed for each type of preoperative ureteral dilation, i.e., pre-stenting and drugs. In multi-arm studies, each pairwise comparison was analyzed separately, with shared intervention groups proportionally divided among the comparisons [13]. For dichotomous outcomes, the number of events and the total number of patients would be divided. For continuous outcomes, only the total number of participants would be divided, and the means and standard deviations left unchanged [13]. The mean and variance from a sample's median, range, and size were estimated according to Hozo’s formula [14].

Study heterogeneity was defined as an I^2^ value. Substantial heterogeneity was defined as an I^2^ value >50%. Meta-analysis was performed using Review Manager (RevMan 5.4) software by Cochrane Collaboration. The quality assessment and publication bias of the included RCTs was performed using the Cochrane Risk of Bias tool RoB 2 and funnel plots (Supplementary figure) [15]. Finally, Grading of Recommendations Assessment, Development, and Evaluation (GRADE) framework used to rate the quality of evidence and strength of recommendations.

## Results

**Fig. 1:**
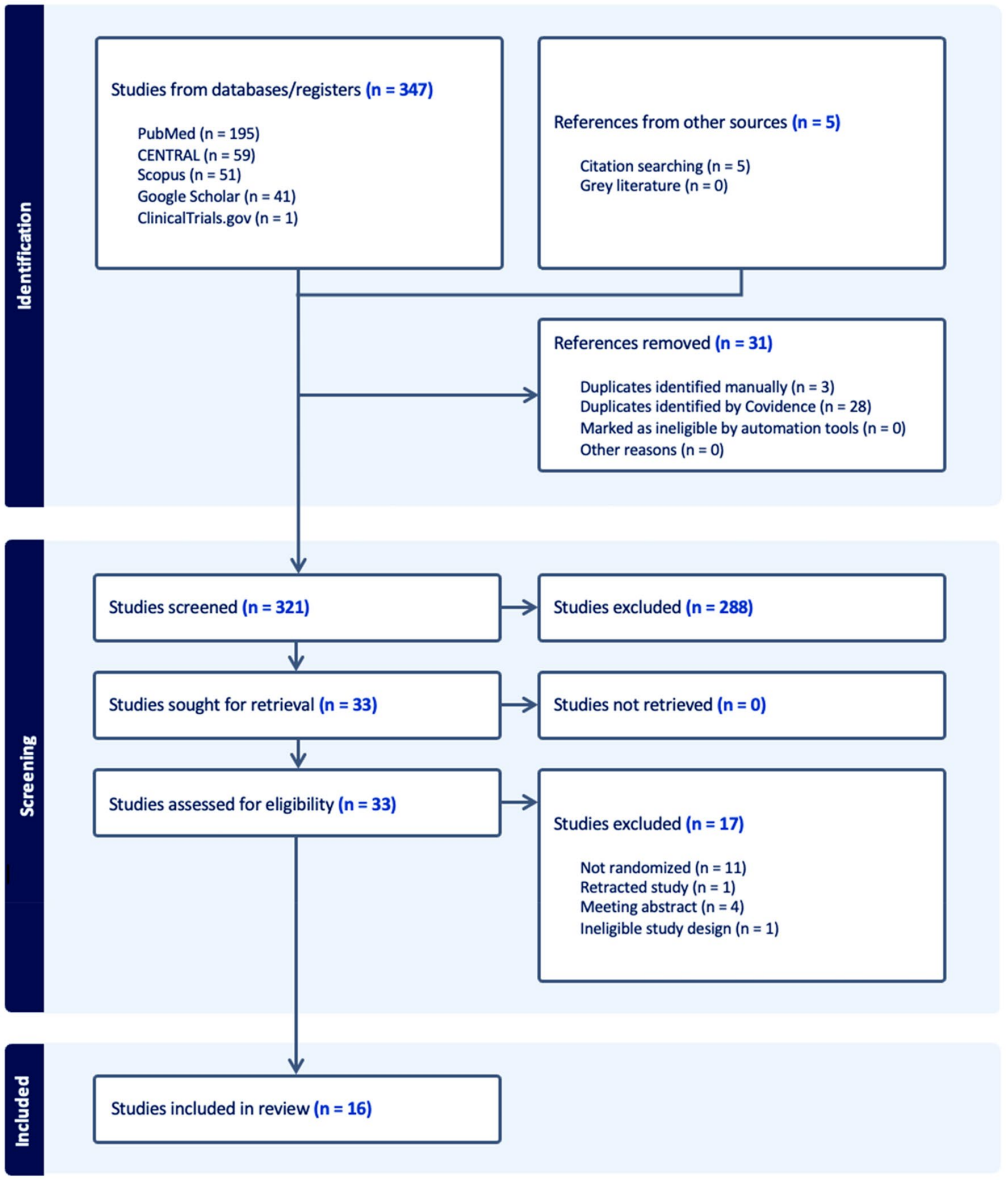
PRISMA flow diagram

A literature search retrieved 352 papers. Thirty-one duplicates were deleted, leaving 321 papers for screening against the title and abstract. 288 papers were excluded. The full texts of 33 studies were screened, and 17 studies were further excluded. Finally, 16 studies were included in the meta-analysis. Figure 1 shows the PRISMA flow diagram.

### Study characteristics and quality assessment

Study characteristics are summarized in Table 1. Supplementary figure demonstrates the details of the quality assessment of the included studies. Seven studies showed a low overall risk of bias. Seven studies showed some concerns regarding the overall risk of bias. Two studies showed a high overall risk of bias. The most frequent reason for bias was missing outcome data, followed by the randomization process. Supplementary Table 1 shows a summary of findings using the GRADE approach to assess the certainty of evidence. GRADE highlights that: i) the risk of bias is moderate—(some studies had unclear or high risk due to lack of blinding or randomization methods); ii) the imprecision is moderate to serious (some outcomes had small sample sizes or wide confidence intervals); iii) indirectness is not serious (populations, interventions, and outcomes are appropriate for the clinical question); iv) the inconsistency is low (the direction of effect was consistent across studies, with moderate heterogeneity at most).

**Table 1:** Characteristics of included studies

Study	Sample	Arms (number of patients)	Stone location	Size and type of ureteroscope	UAS size	Laser/ Energy used for lithotripsy	Study outcome(s)	Conclusion
Abdelaziz 2017 [16]	98	Tamsulosin 0.4 mg for 1 week (51) No drug (47)	Lower ureter	7.5 Fr semirigid	–	Pneumatic lithotripsy	To evaluate the efficacy of Tamsulosin on URS outcomes	Post-Tamsulosin URS was easier and safer; leading to significantly increased SFR and fewer complications
Aydın 2018 [17]	147	No drug (50) Silodosin 8 mg for 1 day (50) Silodosin 8 mg for 3 days (47)	Ureter	Not reported	–	Not mentioned	To assess the effects of administering silodosin before semi-rigid URS outcomes	The use of silodosin for 3 days before ureteroscopy for ureteral stones increases the rate of access to all ureter stones and decreases the complication rate
Ali 2024 [18]	170	Placebo for 1 week before f-URS and for another 2 weeks after the procedure (85) Tamsulosin 0.4 mg once daily for 1 week before surgery + active dilatation using semirigid scope plus 2 weeks of oral Tamsulosin after the procedure (85)	Kidney	8.4 Fr f-URS	Sheath less	Holmium:YAG laser	To evaluate the effect of using perioperative Tamsulosin and semirigid ureteroscope as dilatation methods before the advancement of f-URS to renal collecting system	Tamsulosin and semirigid ureteroscopy are effective and safe methods of ureteral dilatation before f-URS and are associated with deceased operative times and a higher success rate of f-URS navigation to the kidney at the first surgical attempt
Bhattar 2017 [19]	75	Silodosin 8 mg once daily for 2 weeks (25) Tadalafil 10 mg once daily for 2 weeks (25) Multivitamin as a placebo once daily for 2 weeks (25)	Ureter	8/9.8 Fr semirigid	–	Not mentioned	To assess safety and efficacy of silodosin and tadalafil in dilatation of ureteral orifice, ease of ureteroscopic negotiation, operating time, procedural complications and drug related side effects	Both drugs relax ureteral smooth muscle and aid in forward propagation of large size ureteroscope without any significant risk of mucosal injury, hematuria and ureteral perforation with shorter operative time. Drug related side effects were more significant in tadalafil group as compared to silodosin group
Dermir 2022 [20]	137	Tamsulosin 0.4 mg for 7 days (67) No drug (70)	Ureter	8/9.8 Fr semirigid	–	Holmium:YAG laser	To investigate the effect of Tamsulosin use before URS on the success (no residual stone >3 mm) of the operation, and intraoperative and postoperative complication rate	Preoperative use of tamsulosin reduces intra-operative and postoperative complications and improves SFR
Diab 2024 [21]	140	Silodosin 8 mg for 1 week (70) Placebo (70)	Kidney & upper ureter	8.5 Fr f-URS	12–14 Fr	Laser (type not mentioned)	To assess if preoperative administration of silodosin can facilitate the placement of UAS prior to f-URS and reduce the occurrence of ureteric injury in challenging cases	Preoperative silodosin proved effective in preventing significant ureteral wall injury and reducing acute postoperative pain
Elmoazen 2021 [22]	60	Preoperative stenting 2 weeks before URS (20) Tamsulosin 0.4 mg once daily for 1 week before URS (20) Direct URS (20)	Upper & middle ureter	7.5 Fr semirigid	–	Pneumatic lithotripsy	To compare the safety and efficacy of preoperative stenting versus preoperative Tamsulosin versus URS without preoperative treatment in the ureteroscopic management of single upper or middle ureteral stone <20 mm	Preoperative Tamsulosin or stenting before semirigid URS is safe and effective more than direct URS. Preoperative Tamsulosin significantly reduced operative time and postoperative colic. While preoperative ureteral stenting significantly improved stone-free rates, success rates, ureteroscopic access and hospitalization time, and need for ureteral dilatation and auxiliary procedures
Goyal 2021[23]	318	Silodosin 8 mg for 10 days before URS (84) Tamsulosin 0.4mg for 10 days before URS (93) Placebo (multivitamins supplementation) for 10 days before URS (141)	Lower ureter	8/9.8 Fr semirigid	–	Pneumatic lithotripsy	To compare ease of negotiation of ureteroscope at vesicoureteric junction in patients who had received preoperative Tamsulosin vs Silodosin vs no alpha blockers	Alpha blockers are effective, economical and safe preoperatively for URS with 8/9.8 Fr ureteroscope without dilatation. Both drugs are almost equal in results
Kim 2022 [24]	87	Silodosin 8 mg for 3 days before URS (43) Placebo (44)	Kidney & upper ureter	8.5 Fr f-URS	11–13 Fr	Not mentioned	To investigate the effect of Silodosin on preventing ureteral wall injury during UAS insertion and its impact on perioperative outcomes	Preoperative Silodosin medication for just 3 d prevented significant ureteral injury and decreased acute postoperative pain after the RIRS procedure. Silodosin premedication in young patients might more effectively prevent significant ureteral wall injury relating to UAS
Koo 2017 [25]	83	Tamsulosin 0.4 mg daily 7 days before surgery (42) No drug (41)	UPJ & renal pelvis	f-URS	12–14 Fr	None	To investigate the efficacy of preoperative a-blockade to reduce ureteral access sheath insertion force and determine the upper limit required to avoid ureteral injury	Preoperative a-blockade and slow sheath placement may reduce maximal ureteral access sheath insertion force
Köprü 2020 [26]	76	Daily 8 mg silodosin for 10 days (38) No drug (38)	Kidney	7.5 Fr f-URS	9.5 Fr	Laser (type not mentioned)	To evaluate the effect of silodosin on stages of the f-URS	Preoperative use of silodosin facilitated only an insignificant positive effect on UAS placement failure, it eased the f-URS procedure by reducing the entrance to bladder time, entrance to ureteric orifice time and application of UAS time
Lubana 2024 [27]	100	10 ml of local aminophylline (50) Local saline infusion (50)	Ureter	Not reported	–	Pneumatic lithotripsy and/or laser	To assess the duration of procedure, ease of UAS, requirement of DJ Stent and need of further operative interventions after usage of local aminophylline administration	The use of aminophylline was useful and effective in reducing the need of stents and secondary surgery, decreased pain, and increased success rate
Mohey 2018 [28]	127	Silodosin 8 mg for 10 days before URS (62) Placebo (multivitamins) for 10 days before URS (65)	Lower ureter	8/9.5 Fr semirigid	–	Pneumatic lithotripsy	To evaluate the efficacy of Silodosin on the success rate of semirigid URS for the management of large distal ureteric stones	Silodosin prior to URS management of large distal ureteric stones seems to be associated with better advancing of the ureteroscope to access the stone, shorter procedure time, higher SFR, lower incidence of complications, and lesser need for postoperative analgesia
Nam 2024 [29]	160	Tamsulosin 0.4 mg for 1 week preoperatively and postoperatively until the ureteral stent was removed (40) Tamsulosin 0.4 mg for 1 week preoperatively and a placebo postoperatively until the ureteral stent was removed (43) Placebo for 1 week preoperatively and a Tamsulosin 0.4 mg postoperatively until the ureteral stent was removed (36) Placebo throughout the study period (41)	Kidney	9.9 Fr or 9.7 Fr f-URS	12/14 Fr	Holmium:YAG laser	To investigate the effect of administering Tamsulosin before surgery on the successful insertion of UAS, as well as the impact of preoperative and postoperative Tamsulosin use on symptoms related to the ureteral stent	Preoperative Tamsulosin (enhanced the success rate of UAS insertion during RIRS, with no statistically significant differences in ureteral injury, operative time, or SFR. Preoperative and postoperative Tamsulosin did not significantly affect stent-related symptoms or patient comfort
Shaher 2023 [30]	100	Silodosin 8 mg for 10 days before URS (50) No drug (50)	Kidney & upper ureter	9.7 Fr f-URS	11/13 Fr	Holmium:YAG laser	To evaluate the impact of Silodosin on stages of the f-URS procedures, complications, and SFR	Preoperative silodosin was successful in treating stones resulting in shortening the procedural time, with no impact on SFR or complication rate
Tawfeek 2020 [31]	116	Tamsulosin 0.4 mg per day for 1 week preoperatively, and for 2 weeks postoperatively (58) Placebo (58)	Lower ureter	6.5/9.5 Fr semirigid	–	Holmium:YAG laser	To assess the role of Tamsulosin in non-stented ureteroscopy regarding preoperative ureteric dilatation and its impact on postoperative pain and the need for an analgesic	Perioperative Tamsulosin significantly decreased the need for intraoperative dilatation and operative time, but also leaded to a significant decrease in the development of post-operative lower urinary tract symptoms, post-operative pain and the need for analgesia and hospital stay

*URS* ureteroscopy, *f-URS* flexible ureteroscope, *SFR* stone-free rate, *UAS* ureteral access sheath, *RIRS* retrograde intrarenal surgery, *UPJ* ureteropelvic junction

### Results of meta-analysis

#### Meta-analysis of failure to access upper urinary tract (Fig. 2a)

**Fig. 2:**
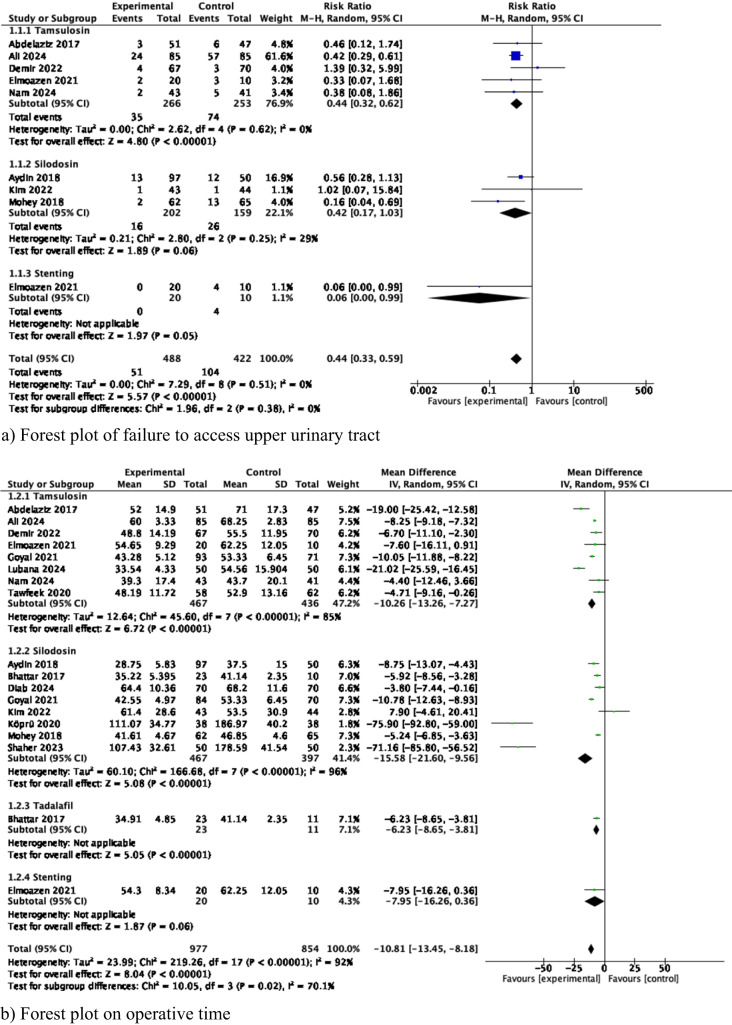

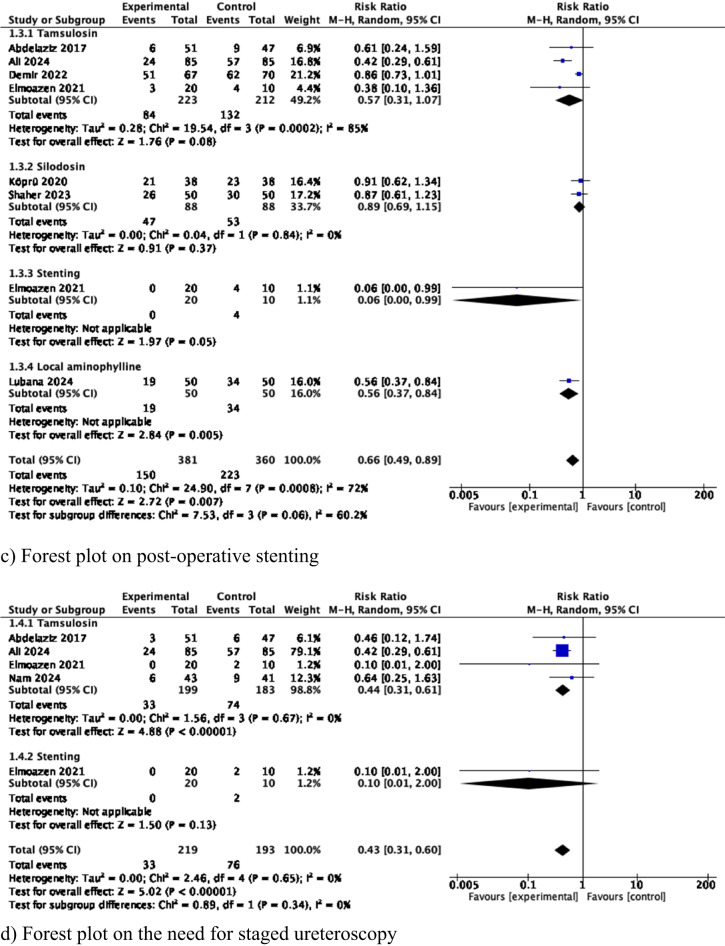

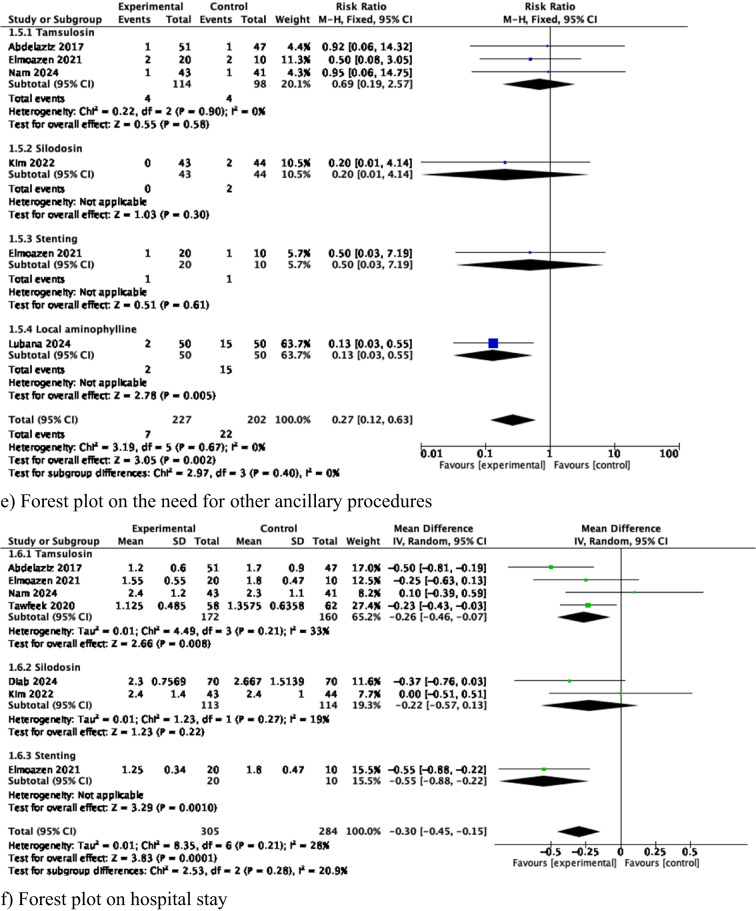

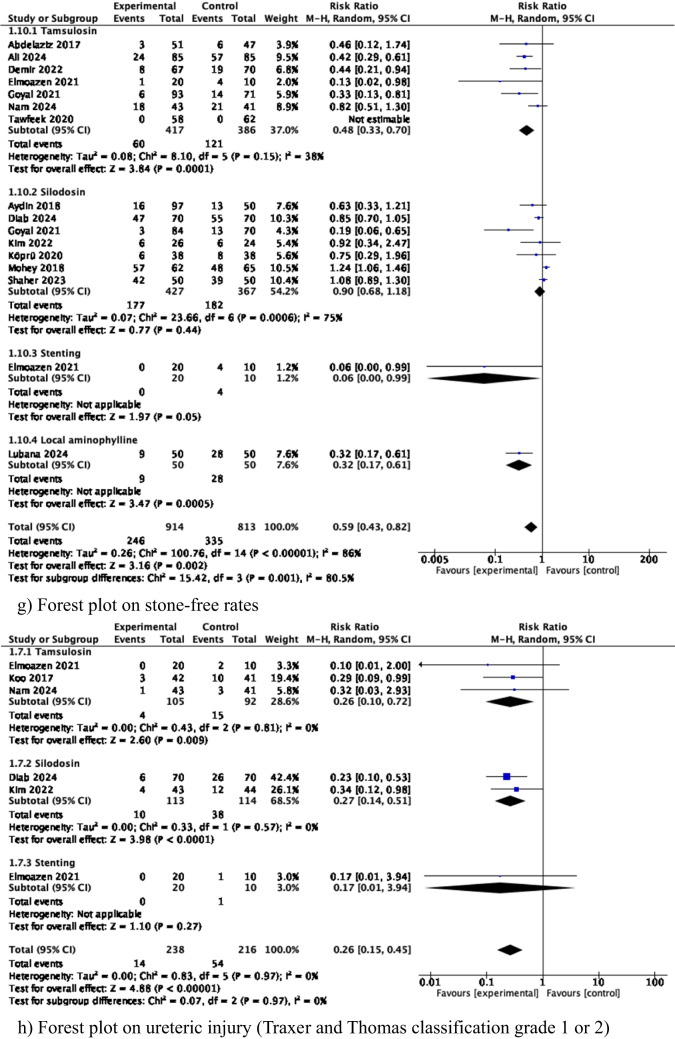

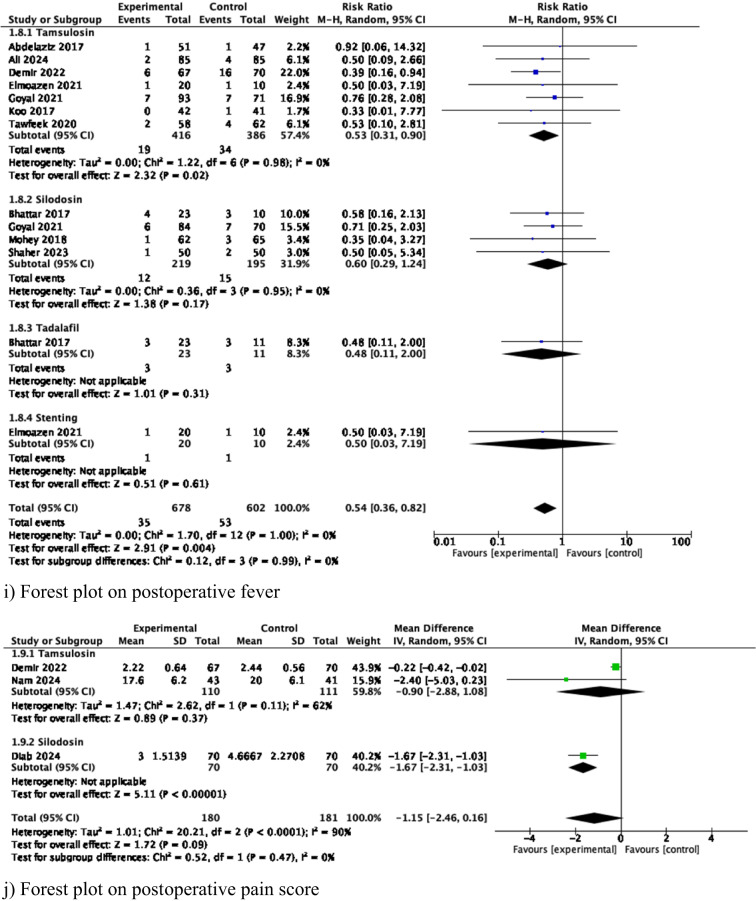
Results of Meta-analysis comparing drug or pre-stenting versus placebo

A meta-analysis from 8 studies (519 cases used Tamsulosin, 361 cases used Silodosin, and 30 used pre-stenting) showed that there were significantly fewer failures in accessing the upper urinary tract in the experimental group (RR 0.44 95% CI 0.33-0.59, *p* < 0.001). Subgroup analysis revealed that failure to access the upper urinary tract was associated with both the way in whether Tamsulosin was administered and also by duration of pre-stenting. There was no heterogeneity among the studies (*I*^2^ 0%).

#### Meta-analysis of operative time (Fig. 2b)

A meta-analysis from 15 studies (903 cases used Tamsulosin, 864 cases used Silodosin, 34 used Tadalafil, 30 used pre-stenting) showed that the mean operative time was significantly shorter in the experimental group compared to control (MD—10.81 min, 95% CI–13.45 to–8.18, *p*<0.001). Subgroup analysis confirmed that mean operative time was significantly in favor of each experimental group. There was considerable heterogeneity among the studies (*I*^2^ 92%).

#### Meta-analysis of postoperative stenting (Fig. 2c)

A meta-analysis from 7 studies (435 cases used Tamsulosin, 176 cases used Silodosin, 30 used stenting, 100 used local Aminophylline) showed that there was less need for postoperative stenting in the experimental group (RR 0.66 95% CI 0.49–0.89, *p* =0.007). Subgroup analysis showed the less need for postoperative stenting was related to the effect of the aminophylline and pre-stenting groups. There was considerable heterogeneity among the studies (*I*^2^ 72%).

#### Meta-analysis of the need for staged ureteroscopy (Fig. 2d)

A meta-analysis from 4 studies (382 cases used Tamsulosin, 30 used stenting) showed that there was significantly less need for staged ureteroscopy in the experimental group (RR 0.43 95% CI 0.31–0.60, *p* < 0.001). Subgroup analysis showed that this was related to the Tamsulosin group. There was no heterogeneity among the studies (*I*^2^ 0%).

#### Meta-analysis of the need for other ancillary procedures (Fig. 2e)

A meta-analysis from 5 studies (212 cases used Tamsulosin, 87 cases used Silodosin and 30 used stenting) showed that there was significantly less need for repeat ureteroscopy or other ancillary procedures (e.g. shockwave lithotripsy, ureterolithotomy) in the experimental group (RR 0.27 95% CI 0.12–0.63, *p* = 0.002). However, subgroup analysis showed this was only related to the local aminophylline group. There was no heterogeneity among the studies (*I*^2^ 0%).

#### Meta-analysis of hospital stay (Fig. 2f)

A meta-analysis from 6 studies (332 cases used Tamsulosin, 227 cases used Silodosin and 30 used stenting) showed that the mean hospital stay was significantly shorter in the experimental group compared to the control (MD—0.3 days, 95% CI–0.45 to–0.15, *p* < 0.001). Subgroup analysis showed that this was related to the Tamsulosin and pre-stenting groups. There was low heterogeneity among the studies (*I*^2^ 28%).

#### Meta-analysis of stone-free rates (Fig. 2g)

A meta-analysis from 14 studies (803 cases used Tamsulosin, 794 cases used Silodosin, 30 used pre-stenting, and 100 cases used local aminophylline) showed that there was significantly higher stone-free rate in the experimental group (RR 0.59 95% CI 0.43–0.82, *p* = 0.002). Subgroup analysis showed this was related to the Tamsulosin, pre-stenting, and local aminophylline groups. There was high heterogeneity among the studies (*I*^2^ 86%).

#### Meta-analysis of Grade 1 and 2 ureteric injury (Fig. 2h)

A meta-analysis from 5 studies (197 cases used Tamsulosin, 227 cases used Silodosin and 30 used stenting) showed that there were significantly fewer Traxer and Thomas classification [3] grade 1 or 2 ureteric injuries in the experimental group (RR 0.26 95% CI 0.15–0.45, *p* < 0.001). Subgroup analysis showed that this was related to both alpha-blocker groups. There was no heterogeneity among the studies (*I*^2^ 0%).

#### Meta-analysis of postoperative fever (Fig. 2i)

A meta-analysis from 10 studies (802 cases used Tamsulosin, 414 used Silodosin, 34 used Tadalafil, and 30 used pre-stenting) showed that there was significantly less postoperative fever in the experimental group (RR 0.54 95% CI 0.36–0.82, *p* =0.004). Subgroup analysis showed that this was related only to the Tamsulosin group. There was no heterogeneity among the studies (*I*^2^ 0%).

#### Meta-analysis of postoperative pain score (Fig. 2j)

A meta-analysis from 3 studies (221 cases used Tamsulosin, 140 cases used Silodosin) showed that the mean postoperative pains score was significantly lower in the experimental group compared to the control (MD—1.15, 95% CI–2.46 to 0.16, *p* = 0.09), and this was related only to the Silodosin group. There was considerable heterogeneity among the studies (*I*^2^ 90%).

## Discussion

In this systematic review and meta-analysis of randomized trials, we found several interesting and important results which can impact daily clinical practice when performing ureteroscopy.

### Upper urinary tract access

Our results showed the use of alpha blockers and pre-stenting increases access to ureter. Most frequently series reported 3 days to 2 weeks use for drugs or stents as pre-procedural dilatation techniques. We could not perform subset analysis to say if stents or drugs is the better modality. Neither are we able to comment which drug is the best albeit Silodosin use is more in recent series and is equally effective like tamsulosin.

### Postoperative stenting, staged procedure, and hospital stay

In our analysis, preoperative use of alpha blockers and pre-stenting demonstrated substantial benefits in reducing the need for postoperative stenting, staged ureteroscopy, and ancillary interventions. Similarly, this reduced hospital stay. Fragmentation of ureteral stones frequently results in localized ureteral wall congestion and edema, which can hinder stone fragment passage and lead to stone impaction or obstruction [32]. Active dilation methods, including ureteral dilators, effectively address these issues by facilitating smoother stone transit. However, these techniques are associated with challenges such as increased procedure and healthcare costs, a higher risk of intraoperative complications, and occasional failure of primary ureteroscopy [33]. In contrast, passive dilation achieved through alpha-blockers provides a safer and more cost-effective alternative, improving patient outcomes with fewer procedural risks. Tamsulosin has been particularly effective in mitigating complications related to post-lithotripsy gravel migration due to its ability to relax the distal ureteral smooth muscle [34].

Elmoazen et al. demonstrated that patients in dilation groups—whether pre-stenting or Tamsulosin—showed reduced reliance on ancillary interventions such as DJ stenting, repeat URS, or extracorporeal shockwave lithotripsy (SWL) [35]. Similarly, pre-stenting was associated with a reduced hospital stay (1.25± 0.34 vs 1.8± 0.47 days) and a reduced need for post-operative stenting.

From the results of this meta-analysis, preoperative ureteral dilation allows a safer procedure and an overall reduced hospital stay. Preoperative stenting has a more significant impact in comparison with preoperative medications regarding parameters like the need for postoperative stenting or ancillary procedures, as stated above. The study by Abdelaziz et al. [16] reports that preoperative Tamsulosin increases the overall success rate of the fURS procedure (94.11% vs. 87.23%; p = 0.045). Conversely, the study by Kim et al. [24] did not find a clear impact of preoperative silodosin on reduced hospital stay (p = 0.972).

### Complications and stone-free rate

UAS placement positively impacts is essential in flexible ureteroscopy, making stone extraction easier [36, 37] and reducing intrarenal pressures [38]. This needs to be balanced with a larger UAS size, possibly causing inadvertent ureteral injury [37–39]. The results of the present study are important in showing preoperative ureteral dilation decreases low-grade ureteric injury, with the potential to decrease complication rates while facilitating stone retrieval and contributing to stone-free outcomes. This will arguably be even more important in the era of flexible and navigable suction ureteric access sheaths (FANS) [40], sheath size choice considerations [41], and larger stones being treated with flexible ureteroscopy with advanced laser technology [42]. Notably, there is decreased postoperative fever with intervention, which could be related to dilation of the ureter [43] and lower intrarenal pressures achieved due to access sheath insertion and maybe to lower intrarenal pressure during lithotripsy [37, 38]. This should be further evaluated. Additionally, quality of life post ureteroscopy is a vital parameter to consider post ureteroscopy [44, 45], with the present review showing less postoperative pain in the experimental group. Given the lifetime risk of stone recurrence, this can be impactful for patient counseling on side effects and the patient experience with ureteroscopy. The overall lower complication rates with preoperative stenting and medications are powerful reasons to consider wider application pre-ureteroscopy and can be considered to form part of routine preoperative counseling for ureteroscopy.

The ultimate aim of ureteroscopy is to attain zero residual fragments, balancing complications within a single-stage setting. Residual fragments have implications, with a review reporting that in patients with dust or residual fragments of 4 mm, 30% would experience symptoms or reintervention within 3 years, and the same proportion would experience spontaneous passage within 2 years [46]. When a more stringent residual fragment size criteria of ≤2 mm is applied, there are lower regrowth rates, complications, and reintervention rates [47]. Therefore, urologists should consider preoperative Tamsulosin and local aminophylline to help improve ureteroscopy success.

## Limitations

Our systematic review and meta-analysis highlight how preoperative ureteral dilation, using alpha-blockers, local aminophylline, and pre-stenting, positively and directly impacts intraoperative and perioperative outcomes of sURS and fURS. The higher stone-free rate is one of the most substantial reasons to consider preoperative ureteral dilation. However, the need for pre-stenting must account for the additional procedural and anesthesia costs and risks, possible stent symptoms, and potential higher risks of perioperative infection [48], even if the incidence of postoperative fever in our analysis was lower in the experimental group.

Perhaps medical therapy is an easier way to attain preoperative ureteral dilation. However, there is wide variability in the studies on the duration that patients need to take medications before ureteroscopy. Additionally, this does not guarantee a successful ureteroscopy outcome. Regardless, all the benefits shown in this review indicate that preoperative ureteral dilation is a significant reason for broader implementation, whether through medications or pre-stenting, in all suitable ureteroscopy patients. With new technology and miniaturization, as well as better laser technology, it will be of interest to evaluate in future studies how preoperative dilation would affect laser ablation efficiency, energy consumption, and associated efficacy in ureteroscopy [49]. That said, we must also acknowledge that the included studies exhibit substantial heterogeneity in terms of interventions and variables, rendering it challenging to draw generalized conclusions.

## Conclusion

Preoperative measures, including alpha-blocker medications and pre-stenting, and intraoperative aminophylline can facilitate ureteral dilation, enhancing ureteroscopy's success for ureteral and kidney stones and reducing complications. These options should be discussed with patients. Before ureteroscopy, a regimen of three to fourteen days of alpha-blockers, such as Tamsulosin or Silodosin, may represent a less invasive choice. Further trials are needed to determine the optimal duration for preoperative ureteral dilation.

## Supplementary Information

Below is the link to the electronic supplementary material.

Supplementary file1 (DOCX 467 KB)

## Data Availability

No datasets were generated or analysed during the current study.

## References

[1] Geraghty RM, Davis NF, Tzelves L et al (2023) Best practice in interventional management of urolithiasis: an update from the European Association of Urology guidelines panel for urolithiasis 2022. Eur Urol Focus 9(1):199–208. 10.1016/j.euf.2022.06.014 35927160 10.1016/j.euf.2022.06.014

[2] Geavlete P, Multescu R, Geavlete B (2014) Pushing the boundaries of ureteroscopy: current status and future perspectives. Nat Rev Urol 11(7):373–82. 10.1038/nrurol.2014.118 24890883 10.1038/nrurol.2014.118

[3] Traxer O, Thomas A (2013) Prospective evaluation and classification of ureteral wall injuries resulting from insertion of a ureteral access sheath during retrograde intrarenal surgery. J Urol 189(2):580–4. 10.1016/j.juro.2012.08.197 22982421 10.1016/j.juro.2012.08.197

[4] Aykanat C, Balci M, Senel C et al (2022) The impact of ureteral access sheath size on perioperative parameters and postoperative ureteral stricture in retrograde intrarenal surgery. J Endourol 36(8):1013–7. 10.1089/end.2021.0751 35229631 10.1089/end.2021.0751

[5] Kuntz NJ, Neisius A, Tsivian M et al (2015) Balloon dilation of the ureter: a contemporary review of outcomes and complications. J Urol 194(2):413–7. 10.1016/j.juro.2015.02.2917 25728906 10.1016/j.juro.2015.02.2917

[6] Mitchell C, Kuebker J, McCormick B et al (2017) Lubriglide sequential ureteral dilators(®): a safe and effective method of ureteral dilation. J Endourol 31(6):573–6. 10.1089/end.2017.0007 28264591 10.1089/end.2017.0007

[7] Hedlund P, Rahardjo HE, Tsikas D, Kuczyk MA, Uckert S (2024) Drugs to affect the smooth musculature of the human ureter - an update with integrated information from basic science to the use in medical expulsion therapy (MET). World J Urol 42(1):654. 10.1007/s00345-024-05368-5 39609287 10.1007/s00345-024-05368-5 PMC11604773

[8] Campschroer T, Zhu Y, Duijvesz D, Grobbee DE, Lock MT (2014) Alpha-blockers as medical expulsive therapy for ureteral stones. Cochrane Database Syst Rev 4:CD008509. 10.1002/14651858.CD008509.pub2 10.1002/14651858.CD008509.pub2 24691989

[9] Hollingsworth JM, Canales BK, Rogers MA et al (2016) Alpha blockers for treatment of ureteric stones: systematic review and meta-analysis. BMJ 355:i6112. 10.1136/bmj.i6112 27908918 10.1136/bmj.i6112 PMC5131734

[10] Barzegarnezhad A, Firouzian A, Emadi SA, Mousanejad N, Bakhshali R (2012) The effects of local administration of aminophylline on transureteral lithotripsy. Adv Urol 2012:727843. 10.1155/2012/727843 23082076 10.1155/2012/727843 PMC3469073

[11] Lumma PP, Schneider P, Strauss A et al (2013) Impact of ureteral stenting prior to ureterorenoscopy on stone-free rates and complications. World J Urol 31(4):855–9. 10.1007/s00345-011-0789-6 22037634 10.1007/s00345-011-0789-6 PMC3732763

[12] Page MJ, McKenzie JE, Bossuyt PM et al (2021) The PRISMA 2020 statement: an updated guideline for reporting systematic reviews. BMJ 372:n71. 10.1136/bmj.n71 33782057 10.1136/bmj.n71 PMC8005924

[13] Axon E, Dwan K, Richardson R (2023) Multiarm studies and how to handle them in a meta-analysis: a tutorial. Cochrane Evid Synth Methods 1(10):e12033. 10.1002/cesm.12033 40476010 10.1002/cesm.12033 PMC11795958

[14] Hozo SP, Djulbegovic B, Hozo I (2005) Estimating the mean and variance from the median, range, and the size of a sample. BMC Med Res Methodol 5:13. 10.1186/1471-2288-5-13 15840177 10.1186/1471-2288-5-13 PMC1097734

[15] Higgins JP, Altman DG, Gotzsche PC et al (2011) The cochrane collaboration’s tool for assessing risk of bias in randomised trials. BMJ 343:d5928. 10.1136/bmj.d5928 22008217 10.1136/bmj.d5928 PMC3196245

[16] Abdelaziz AS, Kidder AM (2017) Tamsulosin therapy improved the outcome of ureterorenoscopy for lower ureteral stones: a prospective, randomised, controlled, clinical trial. Afr J Urol. 10.1016/j.afju.2015.12.003

[17] Aydin M, Kilinc MF, Yavuz A, Bayar G (2018) Do alpha-1 antagonist medications affect the success of semi-rigid ureteroscopy? A prospective, randomised, single-blind, multicentric study. Urolithiasis 46(6):567–72. 10.1007/s00240-017-1026-6 29151116 10.1007/s00240-017-1026-6

[18] Ali AI, Abdelfadel A, Rohiem MF, Hassan A (2024) Semirigid ureteroscopy and tamsulosin therapy as dilatation methods before flexible ureteroscopy: evaluation and benefits. World J Urol 42(1):75. 10.1007/s00345-023-04696-2 38329579 10.1007/s00345-023-04696-2 PMC10853079

[19] Bhattar R, Jain V, Tomar V, Yadav SS (2017) Safety and efficacy of silodosin and tadalafil in ease of negotiation of large ureteroscope in the management of ureteral stone: A prosective randomized trial. Turk J Urol 43(4):484–9. 10.5152/tud.2017.83548 29201512 10.5152/tud.2017.83548 PMC5687212

[20] Demir M, Ertas K, Aslan R et al (2022) Does tamsulosin use before ureteroscopy increase the success of the operation? J Coll Physicians Surg Pak 32(2):197–201. 10.29271/jcpsp.2022.02.197 35108791 10.29271/jcpsp.2022.02.197

[21] Diab T, El-Shaer W, Ibrahim S, El-Barky E, Elezz AA (2024) Does preoperative silodosin administration facilitate ureteral dilatation during flexible ureterorenoscopy? A randomized clinical trial. Int Urol Nephrol 56(3):839–46. 10.1007/s11255-023-03824-6 37902925 10.1007/s11255-023-03824-6 PMC10853317

[22] Elmoazen M, Ali Elgabry KFM, Shaker H (2021) Comparative study between preoperative stenting versus preoperative tamsulosin in the ureteroscopic management of upper and middle ureteral stones in adults. Egypt J Surg 40(4):1348–1356. 10.4103/ejs.ejs_231_21

[23] Goyal SK, Gupta MK, Jain N, Sharma B (2021) Does preoperative alpha blocker actually helps in negotiation of 8/9.8Fr ureteroscope through vesicoureteric junction? A single center randomized trial. Global J Res Anal. 10.36106/gjra

[24] Kim JK, Choi CI, Lee SH et al (2022) Silodosin for prevention of ureteral injuries resulting from insertion of a ureteral access sheath: a randomized controlled trial. Eur Urol Focus 8(2):572–9. 10.1016/j.euf.2021.03.009 33741297 10.1016/j.euf.2021.03.009

[25] Koo KC, Yoon JH, Park NC et al (2018) The impact of preoperative α-adrenergic antagonists on ureteral access sheath insertion force and the upper limit of force required to avoid ureteral mucosal injury: a randomized controlled study. J Urol 199(6):1622–30. 10.1016/j.juro.2017.09.173 29410081 10.1016/j.juro.2017.09.173

[26] Kopru B, Ebiloglu T, Kaya E et al (2020) Does preoperative use of silodosin affect the stages of F-URS procedure? Arch Esp Urol 73(1):47–53 31950923

[27] Lubana AS, Priyadarshi S, Sharma G et al (2024) Effects of administration of local aminophylline on patients undergoing ureteroscopic lithotripsy. Urologia 91(3):538–42. 10.1177/03915603231216141 38041571 10.1177/03915603231216141

[28] Mohey A, Gharib TM, Alazaby H et al (2018) Efficacy of silodosin on the outcome of semi-rigid ureteroscopy for the management of large distal ureteric stones: blinded randomised trial. Arab J Urol 16(4):422–8. 10.1016/j.aju.2018.07.002 30534442 10.1016/j.aju.2018.07.002 PMC6277265

[29] Nam KH, Suh J, Shin JH, Chae HK, Park HK (2024) Effect of perioperative tamsulosin on successful ureteral access sheath placement and stent-related symptom relief: a double-blinded, randomized, placebo-controlled study. Investig Clin Urol 65(4):342–50. 10.4111/icu.20240005 38978214 10.4111/icu.20240005 PMC11231658

[30] Shaher H, Sebaey A, Albaky AMA, Mahmoud MAA, Elaal AMA (2023) Efficacy of pre-operative silodosin on flexible ureteroscopy procedure: a randomized controlled study. Arab J Urol 21(4):267–72. 10.1080/2090598X.2023.2208790 38178945 10.1080/2090598X.2023.2208790 PMC10763580

[31] Tawfeek AM, Abdelwahab MS, Higazy A et al (2020) Effect of perioperative selective alpha-1 blockers in non-stented ureteroscopic laser lithotripsy for ureteric stones: a randomized controlled trial. Cent Eur J Urol 73(4):520–5. 10.5173/ceju.2020.0258 10.5173/ceju.2020.0258 PMC7848841 33552579

[32] De Coninck V, Keller EX, Somani B et al (2020) Complications of ureteroscopy: a complete overview. World J Urol 38(9):2147–66. 10.1007/s00345-019-03012-1 31748953 10.1007/s00345-019-03012-1

[33] Mitchell C, Kuebker J, McCormick B et al (2017) Lubriglide sequential ureteral dilators((R)): a safe and effective method of ureteral dilation. J Endourol 31(6):573–6. 10.1089/end.2017.0007 28264591 10.1089/end.2017.0007

[34] Tzortzis V, Mamoulakis C, Rioja J et al (2009) Medical expulsive therapy for distal ureteral stones. Drugs 69(6):677–92. 10.2165/00003495-200969060-00003 19405549 10.2165/00003495-200969060-00003

[35] Elmoazen M, Ali E, Khaled FMB, Shaker H (2021) Comparative study between preoperative stenting versus preoperative tamsulosin in the ureteroscopic management of upper and middle ureteral stones in adults. Egypt J Surg 40(4):1348–1356. 10.4103/ejs.ejs_231_21

[36] Lima A, Reeves T, Geraghty R et al (2020) Impact of ureteral access sheath on renal stone treatment: prospective comparative non-randomised outcomes over a 7-year period. World J Urol 38(5):1329–33. 10.1007/s00345-019-02878-5 31342247 10.1007/s00345-019-02878-5 PMC7190582

[37] De Coninck V, Keller EX, Rodríguez-Monsalve M et al (2018) Systematic review of ureteral access sheaths: facts and myths. BJU Int 122(6):959–69. 10.1111/bju.14389 29752769 10.1111/bju.14389

[38] De Coninck V, Somani B, Sener ET et al (2022) Ureteral access sheaths and its use in the future: a comprehensive update based on a literature review. J Clin Med. 10.3390/jcm11175128 36079058 10.3390/jcm11175128 PMC9456781

[39] Traxer O, Thomas A (2013) Prospective evaluation and classification of ureteral wall injuries resulting from insertion of a ureteral access sheath during retrograde intrarenal surgery. J Urol 189(2):580–4. 10.1016/j.juro.2012.08.197 22982421 10.1016/j.juro.2012.08.197

[40] Gauhar V, Traxer O, Castellani D et al (2024) Could use of a flexible and navigable suction ureteral access sheath be a potential game-changer in retrograde intrarenal surgery? Outcomes at 30 days from a large, prospective, multicenter, real-world study by the European Association of Urology Urolithiasis Section. Eur Urol Focus. 10.1016/j.euf.2024.05.010 38789313 10.1016/j.euf.2024.05.010

[41] Kwok JL, Somani B, Sarica K et al (2024) Multicenter outcome analysis of different sheath sizes for flexible and navigable suction ureteral access sheath (FANS) ureteroscopy: an EAU endourology collaboration with the global FANS study group. Urolithiasis 52(1):162. 10.1007/s00240-024-01662-4 39545972 10.1007/s00240-024-01662-4

[42] Gul T, laymon M, Alrayashi M, Abdelkareem M, Salah M (2024) Successful treatment of staghorn stones with flexible ureteroscopy and thulium fiber laser (TFL) lithotripsy: initial experience with 32 cases. Urolithiasis 52(1):102. 10.1007/s00240-024-01598-9 38937284 10.1007/s00240-024-01598-9 PMC11211131

[43] Herout R, Reicherz A, Lange D, Chew BH (2024) The ureteral response to ureteral stents. The Ureter: A Comprehensive Review. p. 209-19.

[44] Sperling CD, Chelluri R, Dobbs RW et al (2022) Longitudinal changes in quality of life after ureteroscopy for nephrolithiasis. Urology 170:60–5. 10.1016/j.urology.2022.08.030 36058341 10.1016/j.urology.2022.08.030

[45] Ziemba JB, Jones A, Lin G et al (2024) Postoperative recovery of quality-of-life following ureteroscopy for nephrolithiasis: the impact on pain intensity and interference and the ability to participate in social roles. Urology 188:38–45. 10.1016/j.urology.2024.03.019 38508532 10.1016/j.urology.2024.03.019

[46] Tzelves L, Geraghty R, Lombardo R et al (2023) Duration of Follow-up and Timing of Discharge from Imaging Follow-up, in Adult Patients with Urolithiasis After Surgical or Medical Intervention: A Systematic Review and Meta-analysis from the European Association of Urology Guideline Panel on Urolithiasis. Eur Urol Focus 9(1):188–98. 10.1016/j.euf.2022.06.016 35851252 10.1016/j.euf.2022.06.016

[47] Panthier F, Kwok J-L, Tzou DT et al (2024) What is the definition of stone dust and how does it compare with clinically insignificant residual fragments? A comprehensive review. World J Urol 42(1):292. 10.1007/s00345-024-04993-4 38704492 10.1007/s00345-024-04993-4

[48] Corrales M, Sierra A, Doizi S, Traxer O (2022) Risk of sepsis in retrograde intrarenal surgery: a systematic review of the literature. Eur Urol Open Sci 44:84–91. 10.1016/j.euros.2022.08.008 36071820 10.1016/j.euros.2022.08.008 PMC9442387

[49] Kwok J-L, De Coninck V, Ventimiglia E et al (2024) Laser ablation efficiency, laser ablation speed, and laser energy consumption during lithotripsy: what are they and how are they defined? A systematic review and proposal for a standardized terminology. Eur Urol Focus 10(4):599–611 37940392 10.1016/j.euf.2023.10.004

