# Supplementary material for: The influence of pre-stenting and drugs on the outcomes of ureteroscopy for kidney and ureteral stone disease: a systematic review and meta-analysis by the EAU Section of Endourology
Source: World J Urol. 2025 Aug 12;43(1):489. doi: 10.1007/s00345-025-05848-2 (PMC12343641; doi:10.1007/s00345-025-05848-2)

**Supplementary Figure.** Risk of bias of the included studies (ROB-2):

1. Review authors’ judgments about each risk of bias item presented as percentages


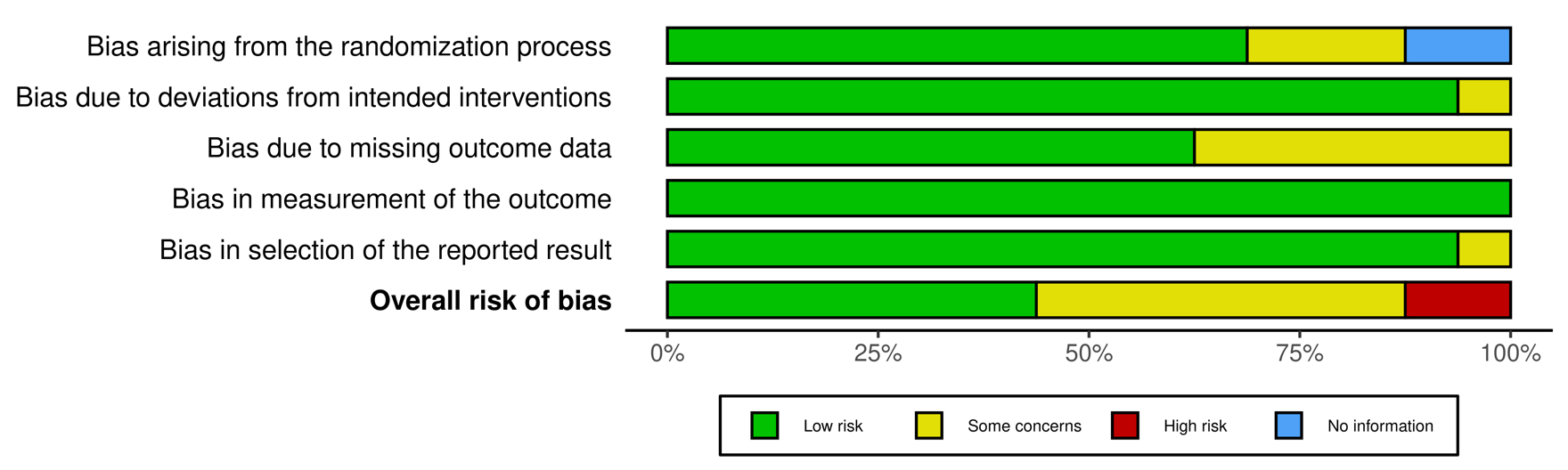


**B)** Review authors’ judgments about each risk of bias item for the included study.


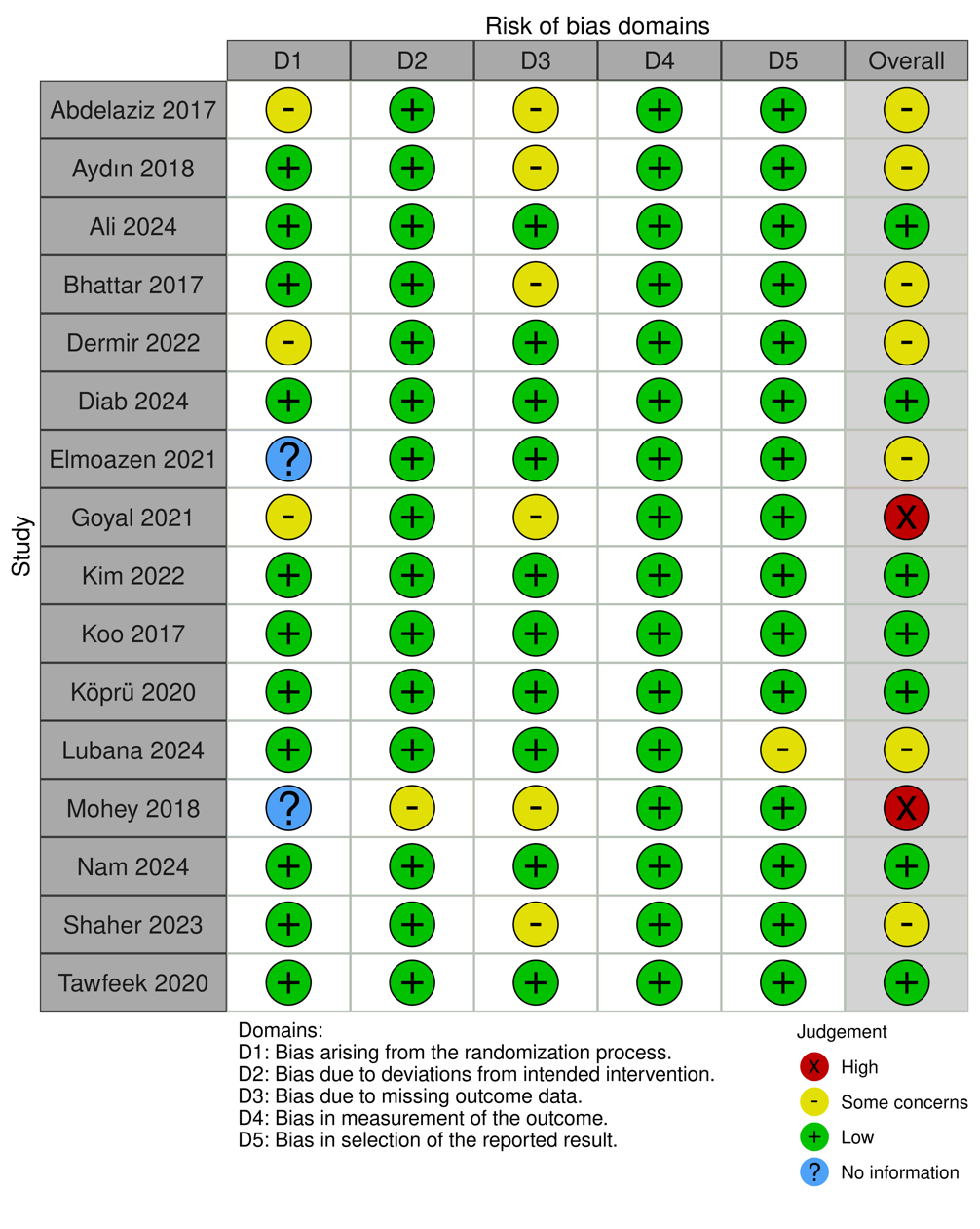

Supplement: Supplementary file 1 — Supplementary file1 (DOCX 467 KB) [file 345_2025_5848_MOESM1_ESM.docx]
